# Removal Performance of KOH-Modified Biochar from Tropical Biomass on Tetracycline and Cr(VI)

**DOI:** 10.3390/ma16113994

**Published:** 2023-05-26

**Authors:** Qingxiang Wang, Yan Yue, Wenfei Liu, Qing Liu, Yu Song, Chengjun Ge, Hongfang Ma

**Affiliations:** 1 School of Environmental Science and Engineering, Qilu University of Technology (Shandong Academy of Sciences), Jinan 250353, China; wangqx0426@163.com (Q.W.); 18765031756@163.com (Q.L.); 1043119575@stu.qlu.edu.cn (Y.S.); 2 Engineering and Technology Center of Electrochemistry, School of Chemistry and Chemical Engineering, Qilu University of Technology (Shandong Academy of Sciences), Jinan 250353, China; yueyan@qlu.edu.cn; 3 Department of Chemistry and Biochemistry, University of California, Los Angeles, CA 90095, USA; wenfei95@gmail.com; 4 Key Laboratory of Agro-Forestry Environmental Processes and Ecological Regulation of Hainan Province, Hainan University, Haikou 570228, China

**Keywords:** KOH-modified biochar, coexisting pollutant, tropical biomass, removal mechanism

## Abstract

Biochar can be used to address the excessive use of tetracycline and micronutrient chromium (Cr) in wastewater that potentially threatens human health. However, there is little information about how the biochar, made from different tropical biomass, facilitates tetracycline and hexavalent chromium (Cr(VI)) removal from aqueous solution. In this study, biochar was prepared from cassava stalk, rubber wood and sugarcane bagasse, then further modified with KOH to remove tetracycline and Cr(VI). Results showed that pore characteristics and redox capacity of biochar were improved after modification. KOH-modified rubber wood biochar had the highest removal of tetracycline and Cr(VI), 1.85 times and 6 times higher than unmodified biochar. Tetracycline and Cr(VI) can be removed by electrostatic adsorption, reduction reaction, π–π stacking interaction, hydrogen bonding, pore filling effect and surface complexation. These observations will improve the understanding of the simultaneous removal of tetracycline and anionic heavy metals from wastewater.

## 1. Introduction

Tetracycline (TC) is widely used as a feed additive to promote growth, treat disease and prevent infection in the aquaculture industry [1]. Moreover, the trace element chromium (Cr) is also added in the form of chromium picolinate or potassium chromate to ensure nutrient supply [2]. As reported, 25–75% of TC [3] and over 90% of hexavalent chromium (Cr(VI)) [4] are excreted to the environment via metabolism. Notably, Cr(VI) in water can directly poison the nervous system, lungs, liver and other organs of aquatic animals after being absorbed by organisms [5] or it can inhibit plant growth and cause metabolic disorder after being absorbed by plants. Although the toxicity of TC in the environment on the human body is not fully understood, it has been reported that TC can induce genetic resistance for microorganisms [6]. Especially, TC and Cr(VI) ingested through the food chain could potentially threaten human health [7]. Therefore, effective measurements must be taken to eliminate the coexisting pollutants of TC-Cr(VI) in aquaculture to improve water quality, ensure human health and keep ecological balance.

Various methods, such as adsorption, photocatalysis and electrocatalysis, have been taken to remove TC and Cr(VI) from the water environment [2,8,9]. Among them, adsorption is broadly adopted for the removal of pollutants in wastewater because of its low cost and high efficiency [10]. The photocatalyst-generated electrons and holes can be utilized for the reduction of Cr(VI) and the oxidation of tetracycline, respectively. Dai et al. found that in the presence of both tetracycline and Cr(VI), the removal efficiency for each was increased by 1.6 and 1.8 times, respectively, compared to their removal in isolated systems [11]. However, most of the current literature on tetracycline and Cr(VI) removal using adsorption techniques focuses on their independent removal via adsorbents, rather than addressing coexisting contamination problems. Currently, the commonly used adsorbents contain activated carbon and nanocomposites [12,13]. However, the wide application of these materials was restricted due to high cost and technical limitations [14]. Nowadays, biochar as an environmentally friendly material has attracted much attention for removing pollutants in the environment. Adsorption capability of TC is 20.4 mg g^−1^ by biochar according to the research of Nguyen et al. [15], but the contaminants removal capacity of pristine biochar is still low in water [16]. As reported, tetracycline can form complexes with heavy metals that exist in cations in aqueous solutions. However, little is known about the interaction of tetracycline with heavy metals present in anionic form in aqueous solutions, as well as the mechanisms through which biochar facilitates their removal.

Chemical modifications, such as acid or alkali impregnation, and element doping [17,18], are developed to improve specific surface area (SSA), density of micropores, and surface functional groups of biochar to enhance its contaminants removal [19]. It was reported that KOH-modified biochar from corn straw has a higher removal capacity of Cr(VI) up to 175.44 mg g^−1^ [18]. Carrying on this study, the research group wants to further explore the improvement of removal capacity of TC and Cr(VI) by KOH-modified biochar.

Additionally, studies have demonstrated that the product yield, graphite carbon structure and SSA of biochar is dependent on the type of raw material [20]. For example, Yi et al. [21] found that the biochar prepared from biomass with a high percentage of cellulose and a low percentage of ash tends to have a higher removal capacity. Environmental pH can affect the electrostatic interaction between biochar and pollutants because it can affect the surface electrical properties of biochar and the existing forms of pollutants. Moreover, the influence of pyrolysis temperature and pyrolysis time on the graphitization degree and surface functional groups of biochar will also affect the removal of pollutants by biochar.

Hainan Province, located in a tropic region, has abundant biomass resources because of the weather conditions [22]. Currently, most of the biomass is discarded or incinerated, causing great waste. Therefore, conversion of the biomass into biochar can not only properly utilize the biomass but also benefit environmental protection. Considering that, the removal of TC and Cr(VI) by the KOH-modified biochar from cassava stalk, rubber wood and sugarcane bagasse was compared. This study aimed (1) to compare the differences in physical and chemical properties of various tropical biochar, as well as their KOH modified biochar, (2) to explore the impact of this various tropical biochar on the removal of TC in water, and (3) to clarify the effect of tropical biochar on the removal of the Cr(VI) and TC coexisting system.

## 2. Materials and Methods

### 2.1. Chemicals

Analytical reagents including KOH, HCl, H_2_SO_4_ and H_3_PO_4_ were purchased from Yantai Yuandong Fine Chemicals Co., Ltd. (Shandong, China). Potassium dichromate (K_2_CrO_4_) and 1,5-diphenylcarbazide was supplied by Sinopharm Chemical Reagent Co., Ltd. (Shanghai, China). Tetracycline hydrochloride and tetracycline hydrochloride standard were obtained from Shanghai Aladdin Reagent Co., Ltd. (Shanghai, China) and the National Institutes for Food and Drug Control, respectively. Chromatographic pure reagents of acetonitrile, formic acid and acetone were from Xilong Science Co., Ltd (Shantou, China).

### 2.2. Biochar

Cassava stalk, rubber wood and sugarcane bagasse were collected from Hainan Province. The biomass was dried and crushed into small pieces of 5 cm and pyrolyzed at 773 K for 2 h in a muffle furnace. The biochar of cassava stalk, rubber wood and sugarcane bagasse were collected and respectively marked as CB, RB and SB.

A certain amount of CB, RB or SB was mixed with 1 mol L^−1^ KOH to ensure the mass ratio of KOH to biochar was 1:1. The mixture was stirred for 30 min and kept for 12 h, and then dried at 378 K. Then, the mixture was pyrolyzed at 1073 K for 2 h in a tubular furnace under the protection of N_2_. The biochar modified with KOH was obtained, and then washed with dilute hydrochloric acid (1 mol L^−1^) and ultrapure water until conductivity less than 10 μS cm^−1^. The washed KOH-modified biochar of CB, RB and SB was dried and respectively marked as MCB, MRB and MSB.

### 2.3. Adsorption Experiments

About 0.1 g of biochar was mixed with 20 mL 10 mg L^−1^ of TC or a mixture of TC and Cr(VI). A control experiment (CK) was set up without the addition of biochar to investigate the interaction between tetracycline and Cr(VI). Adjusting pH values of the mixture to 3, 5, 7, 9 and 11, the solution was filtered to measure the residual TC, total chromium and Cr(VI). Three parallels were set up in all experiments.

We calculated the removal capacity of TC and Cr(VI) based on Equation (1):

(1) $$Q_{e}=(C_{i}-C_{e})V/W$$

Q_e_ (mg g^−1^) represents the ratio of TC and Cr(VI) reduction in solution to biochar mass, C_i_ (mg L^−1^) and C_e_ (mg L^−1^), respectively, represent the original concentration and equilibrium concentration of TC and Cr(VI), V (L) is the volume of wastewater, W (g) is the weight of biochar.

### 2.4. Analytical Methods

Field emission scanning electron microscopy (SEM, Regulus 8100, Tokyo, Japan) was used to measure the morphology of biochar. The settings used for this analysis were a voltage of 15 kV and an electron beam current of 10 μA. The pore size distribution and the SSA of biochar were estimated by a physical adsorption–desorption instrument (Autosorb-IQ, Boynton Beach, FL, USA). These measurements were based on the nitrogen adsorption and desorption curves. The total specific surface area was calculated using the Brunauer–Emmett–Teller (BET) model, the micropore area was determined using the t-plot model, and the micropore volume was estimated using the Horvath–Kawazoe (HK) model. Fourier transform infrared spectroscopy (FTIR, IRAffinity-1s, Tokyo, Japan) was measured with a scanning range of 400–4000 cm^−1^, a scanning frequency of 32, a resolution of 4 and a moving mirror speed of 0.4747. The chemical states of biochar elements (C, N, O and Cr) were measured by X-ray photoelectron spectroscopy (XPS, ESCALAB 250XI, Waltham, MA, USA). The isoelectric point of biochar was tested by a Zeta potentiometer (Zetasizer Nano ZS90, Marvin, UK), with the temperature set to 298 K, an equilibrium time of 120 s and the sample cell being DTS1060.

The total chromium and Cr(VI) were respectively analyzed by inductively coupled plasma atomic emission spectrometry (ICP-AES 9800, Kyoto, Japan) and UV-vis spectrophotometer (UV-5200 PC, Shanghai, China). The residual TC was determined by high-performance liquid chromatography (Shimadzu LC-20 AT, Kyoto, Japan). The chromatographic column was a ShimNex CS C18 (5 μm, 4.6 × 250 mm) (ShimNex CS C18, Suzhou, China). Liquid chromatography conditions were as follows: injection volume of 20 μL, the total flow rate of the mobile phase including 0.1% formic acid (A) and acetonitrile (B) is 1 mL min^−1^, with a column temperature of 313 K, and the operation time of the UV detector is 15 min. The concentration schedule of mobile phases A and B is shown in the supplementary material (Table S1).

### 2.5. Statistical Analysis

Three parallel groups of pollutant removal experiments were set up, and the data were compared by one-way ANOVA.

## 3. Results and Discussion

### 3.1. Characters of Biochar Derived from Cassava Stalk, Rubber Wood and Sugarcane Bagasse

**Figure 1:**
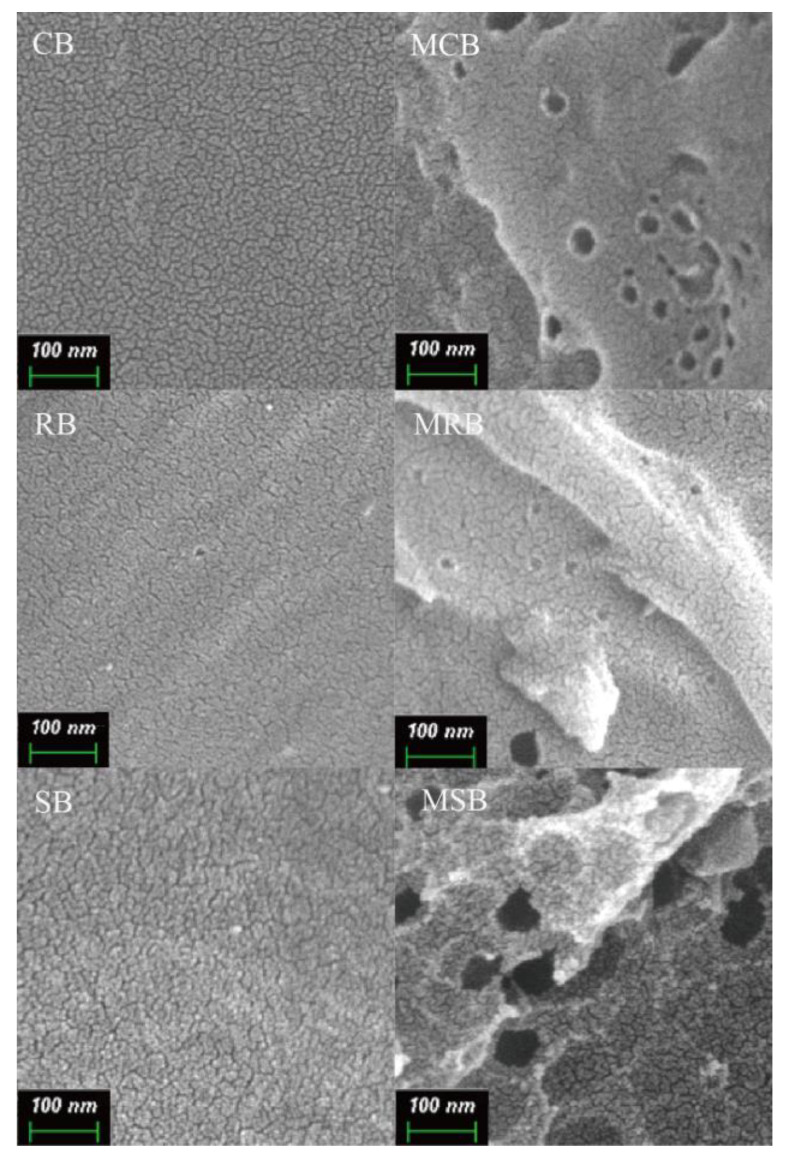
Scanning electron microscopy (SEM) images of cassava stalk biochar (CB), KOH-modified CB (MCB), rubber wood biochar (RB), KOH-modified RB (MRB), sugarcane bagasse biochar (SB) and KOH-modified SB (MSB).

Scanning electron microscopy (SEM) images of cassava stalk biochar (CB), KOH-modified CB (MCB), rubber wood biochar (RB), KOH-modified RB (MRB), sugarcane bagasse biochar (SB) and KOH-modified SB (MSB) were shown in Figure 1. The surfaces of CB, RB and SB were nonporous, while the surfaces of CMB, RMB and SMB were multihole. The different pore characteristics could be attributed to the H_2_, CO and CO_2_ generated from a series of reactions between KOH and carbon materials [23]. As the gases escaped, pores were generated on the surface of the biochar [24]. Compared to MCB and MRB, pores formed on MSB had a larger size as the intermediates from lignin were further decomposed to form pores during the second pyrolysis process [25]. Notably, the lignin content of sugarcane bagasse (28.45%) was the highest compared to those of rubber wood (13.90%) and cassava stalk (16.95%) (Table 1). Moreover, KOH modification can significantly improve the micropore volume of biochar [26].

**Figure 2:**
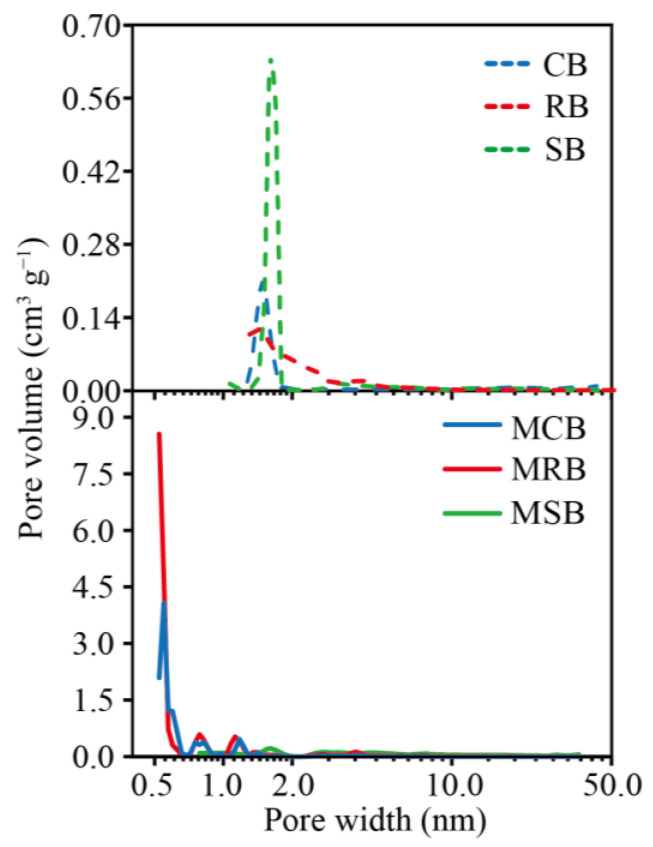
Pore diameter distribution of CB and MCB, RB and MRB, SB and MSB.

As a result, after KOH modification, the nitrogen adsorption capacity of biochar is greatly increased (Supplementary Materials, Figure S1), the SSA of CB and RB each increased 93.6 times and 6.9 times (Table 2). For example, the percentage of micropore surface area of CB was 7.23%, while it increased to 88.92% for MCB. The percentage of micropore volume of CB was 0.00%, while it increased to 75.95% for MCB. Similar results were also obtained for RB and MRB (Table 2). Meanwhile, the pore size of KOH-modified biochar was much smaller (<1 nm) (Figure 2). An et al. [27] had similar findings. Notably, the SSA of SB had no significant change after KOH modification (Table 2). The reason could be that some micropores collapsed and merged into mesopores during the pyrolysis process.

**Figure 3:**
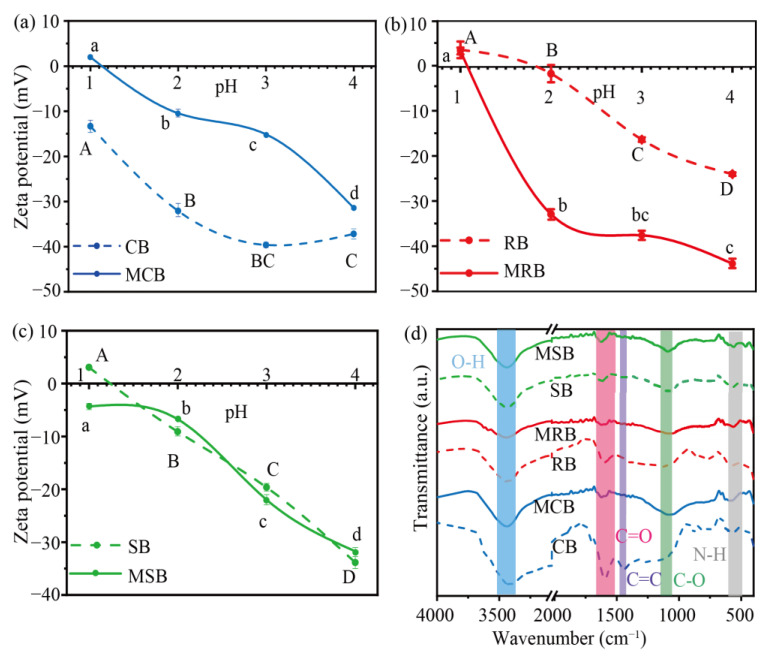
Zeta potential (**a**–**c**) and Fourier-transform infrared spectroscopy (FTIR) (**d**) of CB, MCB, RB, MRB, S and MSB. (The letters indicate a significant difference at *p* < 0.05 in the zeta potential at different pH, with uppercase letters corresponding to pristine biochar and lowercase letters corresponding to the KOH modified biochar, respectively).

The zeta potential of biochar tends to decrease as the environmental pH increases (Figure 3a–c) for the enhanced deprotonation of functional groups on the biochar surface [28]. In addition, the isoelectric points varied with different biochar types (Figure 3a–c). In measurements, both CB and MSB were negatively charged, and the isoelectric points of MCB, RB, MRB and SB were pH = 1.1, 1.8, 1.1 and 1.2. The increased isoelectric point of MCB possibly resulted from the reaction between KOH and acid functional groups of biochar [29]. However, the decreased isoelectric point of MRB and MSB was attributed to more negative charges produced on the biochar surface after KOH modification [30].

Biochar derived from cassava stalk, rubber wood and sugarcane bagasse had similar functional groups in our study, and the proposed chemical structure of these biochar is shown in Figure S2 [31]: –OH of alcohol or phenolic [18], C–H of alkane [32], C=O of ester or aldehyde [33], C=C of aromatic structure [34] and C–O–C of ether [35] (Figure 3d). The functional groups are formed by cellulose, hemicellulose and lignin at 673–773 K [36]. The surface functional groups of the biochar have not changed after being modified; the possible reason is that less KOH was used to modify biochar in this study, resulting in little effect on the surface functional groups of biochar. Lv et al. [37] found that a high dosage of KOH (mass ratio of KOH to biochar greater than 4) could convert stable quinone and carboxyl to unstable C–O–C and C–OH during pyrolysis, while low dosage of KOH has no significant impact on biochar functional groups.

### 3.2. Environmental pH Affected the Removal of TC

TC is an amphoteric molecular, and its existing form depends on the environmental pH: TCH^3+^ (pH < 3.30), neutral or amphoteric ions (3.30 < pH < 7.68), TCH^−^ (7.68 < pH < 9.68) and TC^2−^ (pH > 9.68) [38]. The chemical structure of tetracycline is shown in Figure S3 [39].

**Figure 4:**
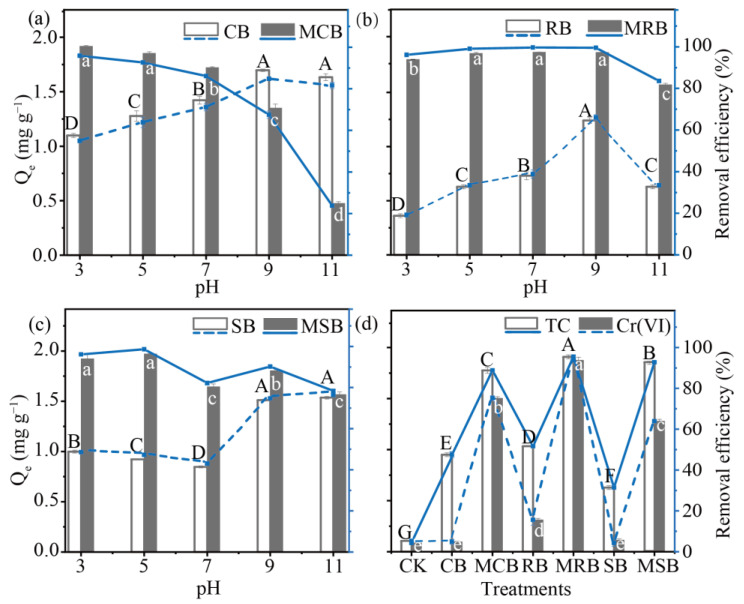
Adsorption capacity for tetracycline under pH = 3, 5, 7, 9 and 11 (**a**–**c**) and for tetracycline (TC) and hexavalent chromium (Cr(VI)) (**d**) of CB, MCB, RB, MRB, SB and MSB. (The letters indicate a significant difference at *p* < 0.05 of different treatments.)

The TC removal capability of tropical biomass biochar and their KOH-modified ones were closely related to the environmental pH values. For example, the amount of TC removed by CB was increased from 1.099 at pH = 3 to 16.330 mg g^−1^ at pH = 11. Conversely, the removed TC by MCB reduced from 1.918 to 0.476 mg g^−1^ due to the increased electrostatic repulsion between TC and MCB (Figure 4a) [40]. MRB showed high removal efficiency (96.11–99.55%) for tetracycline at pH = 3–9, while MCB and MSB showed high removal efficiency within a relatively narrow pH range (pH = 3–5). The biochar’s pore structure can increase tetracycline removal through the intraparticle diffusion process, which can affect the maximum adsorption capacity of the biochar to a certain extent. Consequently, the maximum adsorption capacity of MCB and MRB is higher than that of MSB. However, the tetracycline removal process is not controlled by one pathway, so despite MCB having a slightly larger pore volume, MRB can still exhibit a higher tetracycline removal capacity. For pristine biochar, CB showed a higher tetracycline removal efficiency than both RB and SB at all pH values. Both pristine and KOH-modified biochar showed variations in tetracycline removal efficiency with pH, depending on the used feedstocks.

The TC removed by MCB was significantly higher than that of CB when the solution pH ≤ 7, which was because the major way of removing TC by CB might be hydrogen bond and π–π stacking interaction [41]. Results from FTIR showed that biochar contained –OH and benzene ring, while TC also contained amino and benzene ring. Atomic O of the hydroxyl group in biochar could form hydrogen bond with hydrogen in the amino group of TC. Moreover, π electrons in the benzene ring of TC and biochar could form a π–π stacking interaction through conjugation [42]. Prasannamedha et al. [43] also proved that π–π interactions and hydrogen bonds were essential for sulfamethoxazole removal by sugarcane bagasse biochar. After modification, the impact of pH on the removal of tetracycline by RB and SB also shifted: notably, RB exhibited increased adaptability to pH variations post-modification. The amount of TC removal by RB increased first, followed by a decrease as the pH of solution increased. At pH = 9, the removal capacity of RB reached the maximum, with an efficiency of 1.32 mg g^−1^. The removal efficiency of TC by MRB remained at a high level in a wide pH range (pH = 5, 7 and 9), and the removal efficiency of TC decreased under strong alkali or strong acid conditions, which may be due to the enhancement of intraparticle diffusion by the pores created through the KOH modification process. Similarly, alkaline conditions favor the removal of tetracycline by SB, while acidic conditions favor the removal of tetracycline by MRB. One possible explanation is that KOH modification reduces the number of acidic functional groups in MSB, enhancing its ability to bind TCH^3+^ under acidic conditions.

### 3.3. TC and Cr(VI) Removal Greatly Increased by KOH-Modified Biochar in the Binary System

In the TC-Cr(VI) coexisting system, the removal rate of TC was 5.3%, and that of Cr(VI) was 4.3%, which might be due to the reaction between Cr(VI) and TC. Cr(VI) can be reduced by tetracycline, while tetracycline can be oxidized by Cr(VI) [44]. Remarkably, biochar addition increased TC and Cr(VI) removal (Figure 4d). The removal of TC by CB, RB and SB was each 0.950, 1.033 and 0.628 mg g^−1^, while the removal of TC by MCB, MRB and MSB increased by 86.9%, 84.8% and 194.7%. The increase of TC removal by KOH-modified biochar could be attributed to the complexation reaction between Cr(III) and TC [44]. Moreover, removal rate of Cr(VI) by CB, SB and RB was, respectively, 0.097, 0.114 and 0.312 mg g^−1^, and the larger SSA of RB increased the pollutant removal (Table 2). The removal capacity of Cr(VI) is 0.104, 0.375 and 0.125 mg g^−1^ in a binary system; added with CB, RB and SB, it increased by 14.5, 5.0 and 10.2 times in the corresponding KOH-modified biochar. The removal efficiency of tetracycline (95.5%) and Cr(VI) (94.38%) by MRB was higher than that achieved by MCB and MSB. In a binary system, the removal efficiency of tetracycline by CB, MCB, MRB, SB and MSB decreased compared to a single system, possibly due to competitive adsorption between tetracycline and Cr(VI). However, in the binary system, RB showed an 18.22% increase in tetracycline removal compared to the single system. This may be due to the fact that RB promoted the reaction between tetracycline and Cr(VI), resulting in the formation of Cr(III). RB demonstrated superior removal efficiency for both tetracycline and Cr(VI) in the coexisting system, exceeding CB by 3.18 times and SB by 2.74 times for Cr(VI) removal efficiency. The complexation of Cr(III) with tetracycline promoted the removal of tetracycline. Similar results were reported by Qu et al. [45] and Tian et al. [46].

**Figure 5:**
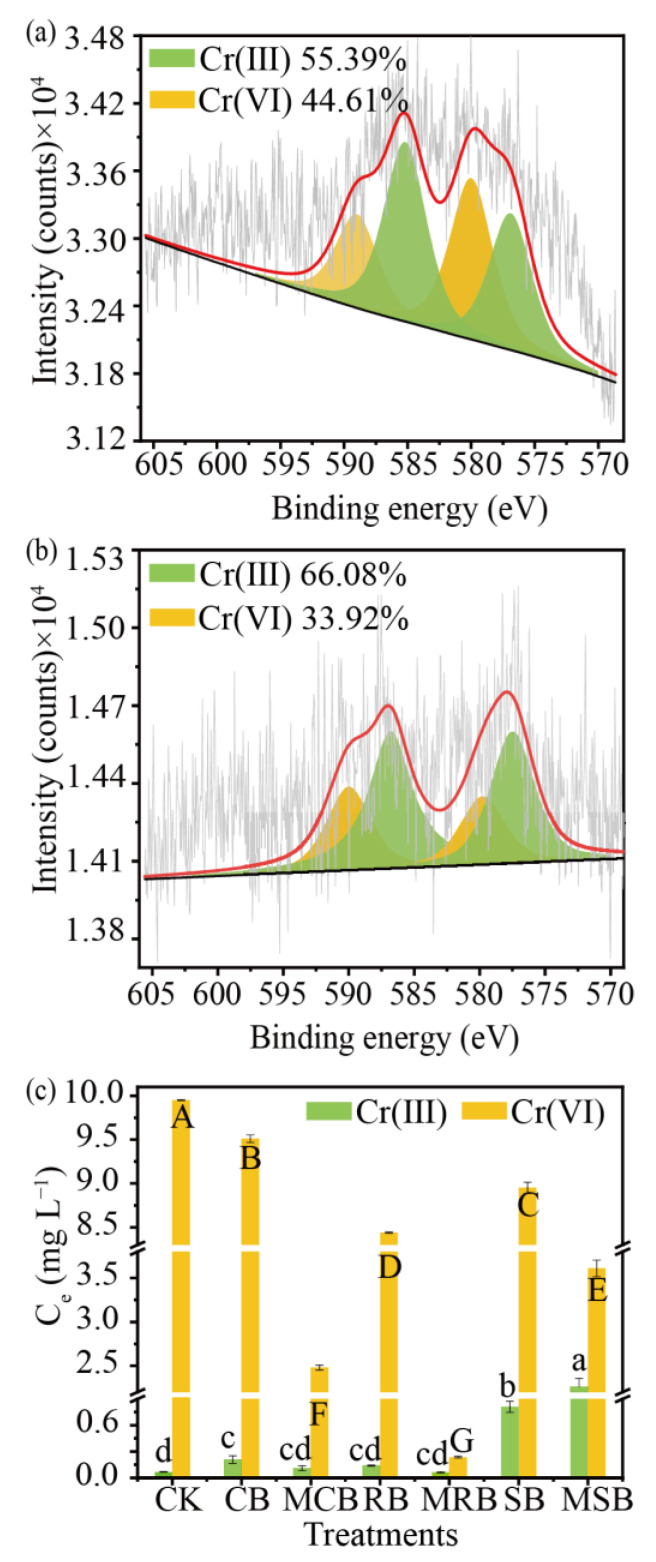
High-resolution XPS spectra of Cr2p in solution (**a**) and on biochar surface (**b**) after MRB treatment, and Cr(III) and Cr(VI) concentration in solution after (**c**) CB, MCB, RB, MRB, SB and MSB treatment. (The letters indicate significant difference at *p* < 0.05 of different treatments).

The concentration of Cr(III) and Cr(VI) was, respectively, 0.05 and 9.95 mg L^−1^ in the binary system, which indicated Cr(VI) could react with TC to generate Cr(III). Moreover, the content of Cr(VI) decreased by 4.40–98.9%, to 9.51–0.062 mg L^−1^, while the content of Cr(III) increased by 0.14–22.10% after biochar addition, which was because the persistent free radicals generated by biochar could act as electron donors to reduce Cr(VI) [47]. The Cr(III) concentration increased to 2.26 mg L^−1^ after MSB treatment in the TC-Cr(VI) coexisting system, which was significantly higher than that after SB addition. Nevertheless, the concentration of Cr(III) in the solution after CB and RB treatment was not significantly different from that after MCB and MRB treatment. The possible reason is that the SSA and pore volume of MCB and MRB are larger than those of MSB, resulting in more active sites and being able to remove more pollutants. Therefore, more Cr(III) was removed by MCB and MRB than MSB, resulting in less Cr(III) in the solution after MCB and MRB treatment (Figure 5c). Moreover, the reduced Cr(III) could be removed by pore filling, especially the micropore that is smaller than 1.27 nm, because TC cannot be removed by those pores [48].

XPS further analyzed the different states of chromium on the surface of MRB and in the solution after the MRB treatment (Figure 5a,b). Based on the results, two characteristic peaks were detected: the peaks situated at 589.0 and 579.8 eV were assigned to Cr2p1/2 and Cr2p3/2 of Cr(VI), and the peaks located at 586.8 and 576.9 eV represent Cr2p1/2 and Cr2p3/2 electron orbitals of Cr(III) [49]. Results from XPS demonstrated that the reduced Cr(VI) enhanced after MSB addition, due to the relative content of Cr(III) in the binary system having increased from 0.50% to 55.39%, and that of Cr(VI) having decreased from 99.5% to 44.61%. In addition, the peaks of Cr(III) (66.08%) and Cr(VI) (33.92%) were also detected on the surface of MSB after TC-Cr(VI) treatment, which indicated that MRB could adsorb Cr(VI) and reduce some Cr(VI) to Cr(III). Meanwhile, the reduced Cr(III) could be fixed on the biochar surface; a similar process was also reported by Bai et al. [50].

Based on the above ICP and XPS results, the adsorption and reduction efficiency of Cr(VI) by MRB were studied. The residual amount of total chromium is 0.126 mg L^−1^ after MRB treatment in the binary system, of which the residual Cr(III) and Cr(VI) was, respectively, 0.066 and 0.060 mg L^−1^. The rest was removed by MRB, and the molar ratio of Cr(III) to Cr(VI) fixed by MRB is about 2:1 (Figure 5b). It is calculated that 66.49% of Cr(VI) was reduced and 32.92% of Cr(VI) was adsorbed by MRB in the coexisting system.

### 3.4. Mechanism of TC and Cr(VI) Removed by Biochar

**Figure 6:**
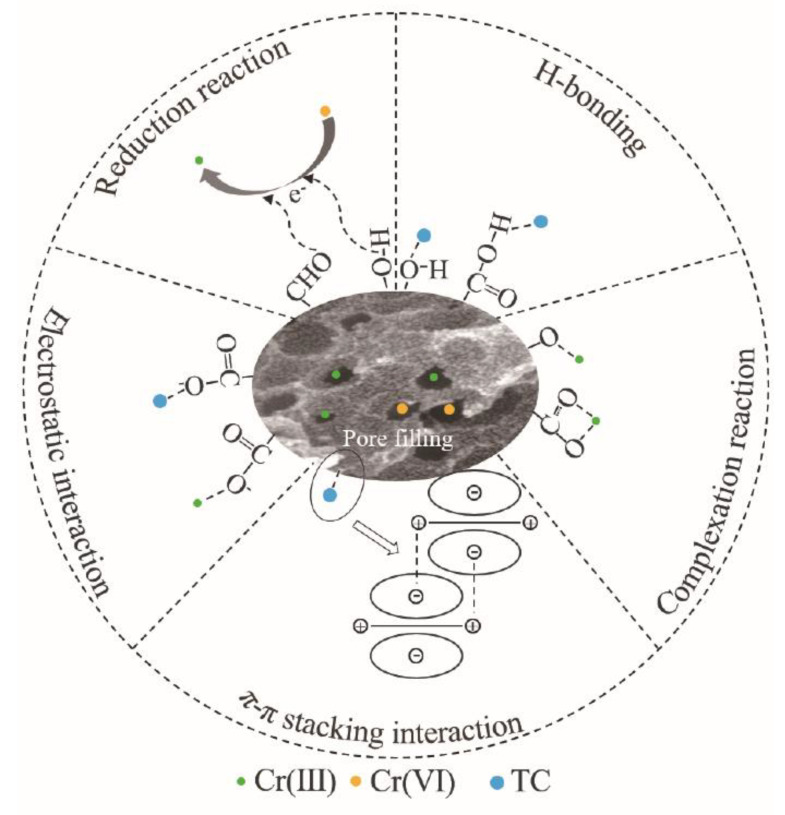
Mechanism of TC-Cr(VI) removal by biochar.

Various mechanisms including π–π stacking interaction, electrostatic interaction, pore filling and hydrogen bonding existed in the TC removal process due to biochar addition (Figure 6). Aromatic rings, –OH and C–O on the surface of biochar can promote the removal of tetracycline through π–π stacking and hydrogen bonding. Furthermore, the pore generated by KOH modification can promote a pore-filling effect. The electrostatic effect can affect the removal of tetracycline by biochar and is especially obvious in the removal of tetracycline by MCB. XPS results demonstrate that Cr(VI) can be immobilized on the surface of biochar by adsorption and can also be reduced by the biochar [45,51]. The key mechanisms for the removal of Cr(VI) and TC by biochar include: (1) biochar facilitating the production of Cr(III) and the formation of complexes with TC, resulting in the simultaneous removal of both tetracycline and hexavalent chromium; (2) larger SSA and pore volume of modified biochar can provide more adsorption sites [52], and these pore structures are conducive to the removal of the TC and Cr(VI) through pore filling.

## 4. Conclusions

Feedstock type affects the physicochemical properties of biochar and tetracycline removal pathways. In the treatment with one pollutant, KOH-modified biochar enhanced tetracycline removal by up to 3.50 times (CB), 4.99 times (RB) and 2.14 times (SB), respectively, compared with the pristine biochar. The tetracycline removal by biochar was significantly affected by pH, with the removal mechanism involving electrostatic adsorption, hydrogen bonding, π–π stacking interactions and complexation effect. MRB displayed better adaptability to pH changes, making tetracycline removal efficiency (99.07–99.66%) have no significant reduction between pH = 5–9. In the binary system, the removal efficiencies of MCB, MRB and MSB for tetracycline were improved by 186.95% (CB), 184.72% (RB) and 295.54% (SB), respectively, compared with the corresponding pristine biochar. Concurrently, the removal effect of Cr(VI) was increased by 15.35 (CB), 6.37 (RB) and 11.21 (SB) times, respectively. MRB removed Cr(VI) through both adsorption and reduction, with these two pathways accounting for 32.92% and 66.49%, respectively.

This study can provide a reference for simultaneous removal of tetracycline and Cr(VI) using adsorption technology. Nevertheless, there is still room for improvement, such as improving the biochar preparation and avoiding secondary pyrolysis, to conserve energy.

## Figures and Tables

**Table 1:** Analysis of different biomass components.

Biomass Type	Cellulose (%)	Hemicellulose (%)	Lignin (%)
Cassava stalk	21.90	20.55	16.95
Rubber wood	46.55	22.05	13.90
Sugarcane bagasse	42.65	20.70	28.45

**Table 2:** Specific surface area and pore volume of different biochar.

Biochar Type	S_BET_ ^1^ (m^2^ g^−1^)	S_mic_ ^2^ (m^2^ g^−1^)	S_mic_/S_BET_ (%)	V_tot_ ^3^ (cm^3^ g^−1^)	V_mic_ ^4^ (cm^3^ g^−1^)	V_mic_/V_tot_ (%)
CB	13.70	0.99	7.23	0.018	0.000	–
MCB	1282.32	1140.29	88.92	0.582	0.442	75.95
RB	142.06	52.90	37.24	0.101	0.018	17.82
MRB	973.46	904.37	92.90	0.420	0.348	82.86
SB	138.22	107.79	77.98	0.074	0.044	59.46
MSB	156.38	43.84	28.03	0.149	0.020	13.42

^1,2^ in the table represents the specific surface area and the microporous area of biochar, similarly, ^3,4^ represents the total pore volume and the microporous volume of biochar; – represents data not available.

## Data Availability

The data that support the findings of this study are available from the corresponding author upon reasonable request.

## Supplementary Materials

The following supporting information can be downloaded at: https://www.mdpi.com/article/10.3390/ma16113994/s1, Figure S1: Nitrogen adsorption–desorption curve of cassava stalk biochar (CB), KOH-modified CB (MCB), rubber wood biochar (RB), KOH-modified RB (MRB), sugarcane bagasse biochar (SB) and KOH-modified SB (MSB), Figure S2: The proposed chemical structure of biochar, Figure S3: Chemical structure of tetracycline; Table S1: Liquid chromatography mobile phase procedure.
