# Supplementary material for: Removal Performance of KOH-Modified Biochar from Tropical Biomass on Tetracycline and Cr(VI)
Source: Materials (Basel). 2023 May 26;16(11):3994. doi: 10.3390/ma16113994 (PMC10254335; doi:10.3390/ma16113994)
Supplement: Supplementary file 1 [file materials-16-03994-s001.zip › materials-2413988-supplementary.pdf]

Supplementary Material

Removal performance of KOH-modified biochar from tropical biomass on tetracycline and Cr(VI)

Qingxiang Wang [1], Yan Yue [2], Wenfei Liu [3], Yu Song [1], Chengjun Ge [4] and Hongfang Ma [1,*]

[1] School of Environmental Science and Engineering, Qilu University of Technology (Shandong Academy of Sciences), Jinan 250353, China
[2] Engineering and Technology Center of Electrochemistry, School of Chemistry and Chemical Engineering, Qilu University of Technology (Shandong Academy of Sciences), Jinan 250353, China
[3] Department of Chemistry and Biochemistry, University of California, Los Angeles, CA 90095, USA
[4] Key Laboratory of Agro-Forestry Environmental Processes and Ecological Regulation of Hainan Province, Hainan University, Haikou 570228, China
* Corresponding author: hongfangma2011@163.com; Tel.: 8615098705577

Table S1 Liquid chromatography mobile phase procedure.

Time (min)	Aqueous phase concentration (%)
0.01	95
2.30	95
8.50	25
9.00	0
11.60	0
11.70	95
15.00	95

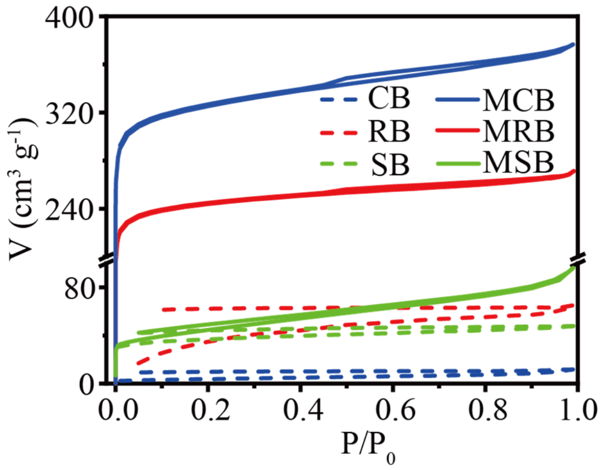

Figure S1 Nitrogen adsorption-desorption curve of cassava stalk biochar (CB), KOH-modified CB (MCB), rubber wood biochar (RB), KOH-modified RB (MRB), bagasse biochar (SB) and KOH-modified SB (MSB).

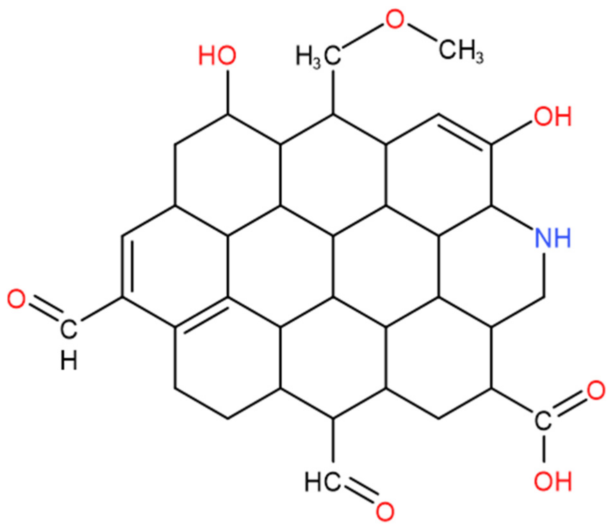

Figure S2. The proposed chemical structure of biochar.

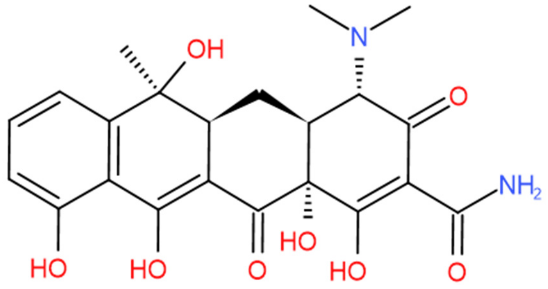

Figure S3. Chemical structure of tetracycline.
